# Seabuckthorn Pulp Oil Protects against Myocardial Ischemia–Reperfusion Injury in Rats through Activation of Akt/eNOS

**DOI:** 10.3389/fphar.2016.00155

**Published:** 2016-06-29

**Authors:** Kapil Suchal, Jagriti Bhatia, Salma Malik, Rajiv Kumar Malhotra, Nanda Gamad, Sameer Goyal, Tapas C. Nag, Dharamvir S. Arya, Shreesh Ojha

**Affiliations:** ^1^Cardiovascular Research Laboratory, Department of Pharmacology, All India Institute of Medical Sciences New Delhi, India; ^2^Department of Pharmacology, R. C. Patel Institute of Pharmaceutical Education and Research Shirpur, India; ^3^Department of Anatomy, All India Institute of Medical Sciences New Delhi, India; ^4^Department of Pharmacology and Therapeutics, College of Medicine and Health Sciences, United Arab Emirates University Abu Dhabi, UAE

**Keywords:** apoptosis, myocardial ischemia–reperfusion injury, inflammation, oxidative stress, seabuckthorn, lehberry, edible oil, natural products

## Abstract

Seabuckthorn (SBT) pulp oil obtained from the fruits of seabuckthorn [*Hippophae rhamnoides* L. (Elaeagnaceae)] has been used traditionally for its medicinal and nutritional properties. However, its role in ischemia–reperfusion (IR) injury of myocardium in rats has not been elucidated so far. The present study reports the cardioprotective effect of SBT pulp oil in IR-induced model of myocardial infarction in rats and underlying mechanism mediating activation of Akt/eNOS signaling pathway. Male albino Wistar rats were orally administered SBT pulp oil (5, 10, and 20 ml/kg/day) or saline for 30 days. On the day 31, ischemia was induced by one-stage ligation of left anterior descending coronary artery for 45 min followed by reperfusion for 60 min. SBT pulp oil pretreatment at the dose of 20 ml/kg observed to stabilize cardiac function and myocardial antioxidants such as glutathione, superoxide dismutase, catalase, and inhibited lipid peroxidation evidenced by reduced malondialdehyde levels as compared to IR-control group. SBT pulp oil also improved hemodynamic and contractile function and decreased tumor necrosis factor and activities of myocyte injury marker enzymes; lactate dehydrogenase and creatine kinase-MB. Additionally, a remarkable rise in expression of pAkt–eNOS, Bcl-2 and decline in expression of IKKβ/NF-κB and Bax was observed in the myocardium. The histopathological and ultrastructural salvage of cardiomyocytes further supports the cardioprotective effect of SBT pulp oil. Based on findings, it can be concluded that SBT pulp oil protects against myocardial IR injury mediating favorable modulation of Akt-eNOS and IKKβ/NF-κB expression.

## Introduction

Inflammation and oxidative stress are the main pathophysiological processes involved in ischemia–reperfusion (IR) injury of myocardium and contribute to increased cardiovascular morbidity and mortality (Yellon and Hausenloy, 2007). Both these mechanisms trigger activation of various signaling pathways including Protein Kinase B (Akt)–endothelial nitric oxide synthase (eNOS) pathway. This Akt–eNOS dynamically participates in regulating IR injury in response to exogenous stimuli. Serine/threonine protein kinase-Akt belongs to a family of signal transduction enzymes that has been implicated in cellular survival through its antioxidant, anti-inflammatory, and anti-apoptotic pathways of protection. Upon activation, Akt translocates from plasma membrane to intracellular compartment and phosphorylates variety of substrates. One of these is eNOS, which is activated through phosphorylation at serine 1177 by Akt. The phosphorylatoin of eNOS leads to increased production of nitric oxide (NO) that suppresses the release of adhesion molecules and cytokines and thus, exerts anti-inflammatory activity (Han et al., 2012; Zhang et al., 2014).

Recently, many investigators have shown the protective effect of various plant products against myocardial IR injury by activating Akt/eNOS-signaling pathway (Han et al., 2012; Pei et al., 2013). In past few years, the plant extracts have also aroused mounting interest in their utilization as dietary food supplements or alternative medicine due to their strong antioxidant activity. The diet rich in natural antioxidants can prevent or delay the incidence of pathological changes due to increased oxidative stress and few investigators have reflected the role of oxidative stress in the pathogenesis of IR injury (Blomhoff, 2005). Although, epidemiological studies indicates a converse relation between intake of plant products and cardiovascular disorders, many people do not consume these products in sufficient amounts and the only feasible means to achieve antioxidants is by the use of dietary supplements (Lampe, 1999). In this regard, fruit extracts act as a concentrated source of antioxidants; lately, attention is being focused on their use to prevent several degenerative diseases accompanied with oxidative stress, inflammation, and cell death.

The fruit extract of seabuckthorn plant [*Hippophae rhamnoides* L. (Elaeagnaceae)], contain many bioactive substances with high medicinal and nutritional potential (Geetha and Asheesh, 2011). Various pharmacological studies on the SBT fruit extracts have documented their anti-apoptotic, antiatherogenic, antidiabetic, antihypertensive, antioxidant, and cardioprotective properties (Geetha et al., 2002, 2009; Basu et al., 2007; Pang, 2008; Zhang et al., 2010; Malik et al., 2011a). Traditionally, the oil extracted from fruits have been used for the treatment of gastric and skin disorders and has nourishing, revitalizing, and restorative action (Yang et al., 2000; Xing et al., 2002). On the basis of the aforementioned facts, we attempted to investigate: (i) the association of IR injury with oxidative stress, inflammation and apoptosis, (ii) whether pretreatment with SBT pulp oil possess any protective effect on these parameters, and (iii) if so, whether this cardioprotective activity in IR injury is mediated by Akt/eNOS pathway.

## Materials and Methods

### Experimental Animals

All the experimental procedures were conducted in accordance with Indian National Science Academy Guidelines for the Care and Use of Animals in Scientific research. The animal study protocol was approved by the Institutional Animal Ethics Committee of All India Institute of Medical Sciences, New Delhi, India (IAEC No. 466/08). Adult male albino Wistar rats aged 6–8 weeks (150–200 g) were used for the study. A total of 72 animals were used in the study. All animals were caged housed at standard laboratory condition in the air conditioned room at temperature 24 ± 1°C, 12 h light/dark cycles with a relative humidity of 50–60%. All animals had free access to standard rodent pellet diet (Ashirwad Industries, Chandigarh, India) and tap water *ad libitum.* They were allowed to acclimatize for 1 week before beginning of the experiment.

### Drugs and Chemicals

The SBT pulp oil was obtained from Mantra Ayurveda, Ghaziabad, India. The commercially available kits for creatine kinase-MB isoenzyme (CK-MB), lactate dehydrogenase (LDH), and tumor necrosis factor-alpha (TNF-α) were obtained from Spinreact, Spain; Logotech private Limited, India and Diaclone Tepnel Company, UK, respectively. All primary antibodies for western blot were procured from Cell Signaling technology, USA except Bax, which was obtained from Abcam, UK. The secondary antibodies were purchased from Merck Genei, India. The kits for biochemical tests such as glutathione (GSH), superoxide dismutase (SOD), and catalase (CAT) standards and thiobarbituric acid standard, i.e., 1,1,3,3-tetraethoxypropane were obtained from Sigma–Aldrich, USA. All other chemicals used in this study were of analytical grade.

### Gas Chromatography–Mass Spectrophotometry (GC–MS) Analysis

#### Sample Preparation

To investigate the composition of SBT pulp oil, GC–MS analysis was performed. To begin with, SBT pulp oil was converted into fatty acid methyl esters using Boron trifluoride/methanol [Association of Official Analytical Chemists (AOAC) official method 969.33]. The samples were filtered through 0.22 μm syringe filter. For GC–MS analysis, the sample (1 μl) was loaded by automatic programmed syringe injector.

#### Chromatographic Conditions Used in GC–MS

To carry out GC–MS analysis of SBT pulp oil, GCMSQP-2010 Plus (Shimadzu, Japan) auto-sampler was used. RTx-5MS column (30 m × 0.25 mm × 0.25 μm) operating in electron impact mode at 70 eV was used to analyze sample in gas chromatograph which was interfaced from a mass spectrometer. The helium gas was used as a carrier at a steady flow rate of 1 ml/min. The column pressure was 81.7 kPa, flow rate 1.21 ml/min and initial column temperature was 80°C (isothermal for 4 min) with gradual increase of 5°C/min to 310°C. A Mass spectrum was prepared at a scan interval of 0.50 s with a mass scan from 40 to 650 *m/z*.

#### Identification of Compounds in SBT Pulp Oil

To interpret GC–MS data, NIST/NIH/EPA Mass spectral database with NIST05 (National Institute of Standards and Technology) MS program v.2.0d and WILEY08 libraries were used. Unknown components were also identified according to their retention time. The names, molecular mass, structure, chemical, and biological activity of identified compound were found using Dr. Duke’s phytochemical and ethnobotanical databases, NCBI-Pubchem, Chem Spider available from Royal Society of Chemistry and various literatures.

### Experimental Groups

Adult, male Wistar albino rats were randomly divided into six experimental groups, each containing 12 rats. The experimental groups were: (I) Sham; (II) IR-control; (III–V) SBT pulp oil treatment groups (5, 10, and 20 ml/kg; p.o.); and (VI) SBT pulp oil *per se* group (20 ml/kg; p.o.). The SBT pulp oil was administered orally to the rats for a period of 30 days while sham and IR-control groups received 3 ml/kg normal saline for the same time period. On day 31, the animals in group (II–V) underwent left anterior descending (LAD) coronary artery occlusion for 45 min and reperfusion for 60 min. Similarly, group I and VI animals underwent the entire surgical procedure except for coronary artery occlusion of LAD.

### Measurement of Hemodynamic Parameters

All experimental animals were anesthetized with intraperitoneal injection of pentobarbitone sodium (60 mg/kg) and ventilated artificially using a positive pressure ventilator (Inco, India). Myocardial ischemia was induced by one stage ligation of LAD coronary artery for 45 min and later reperfused for 60 min. All animals were allowed to stabilize for 15 min before LAD coronary artery occlusion. The hemodynamic parameters such as mean arterial pressures (MAP), heart rate (HR), left ventricular end diastolic pressure (LVEDP), and rate of change of pressure development (±LVd*P*/d*t*) were monitored and recorded at 0, 5, 10, 15, 30, and 45 min during ischemia period and at 0, 5, 10, 15, 30, 45, and 60 min during reperfusion period. At the end of reperfusion period, the animals were sacrificed with an overdose of pentobarbitone sodium (150 mg/kg; i.p.). Blood was withdrawn from the heart and centrifuged at 5000 rpm (Sigma Laborzentrifugen GmbH, Germany) for 20 min to obtain serum for analyzing LDH, CK-MB enzyme activities, and NO and TNF-α levels. Further, the hearts were excised and processed for histopathological, ultrastructural, biochemical, and molecular studies.

### Determination of Mean Area at Risk

At the end of the reperfusion period, monastral blue (0.5 ml/kg) was injected into the left atrium over 30 s to determine the *in vivo* area at risk. After that, animals were sacrificed, and their hearts were excised. Then after, the left ventricle was separated and kept at -20°C for 30 min to allow uniform sectioning. The sections were incubated in 1% buffered TTC pH 8.5 for 20 min at 37°C for visualization of the infarction (Malik et al., 2011b).

### Assessment of Biochemical Parameters

The heart samples stored at -80°C was thawed for biochemical estimation and weighed for tissue homogenization. The tissue homogenate (10%) was prepared from left ventricle in 0.1 M phosphate buffer (pH 7.4) and divided into three parts. One part of this homogenate was used to assess lipid peroxidation by measuring malondialdehyde (MDA) content, the product of lipid peroxidation. The formed MDA in tissues are thiobarbituric acid reacting substances which react with thiobarbituric acid to form color adducts whose absorption was read at 532 nm as described previously (Ohkawa et al., 1979). The second part of the homogenate was centrifuged in equal part of 10% tricarboxylic acid (TCA) at 5000 rpm for 10 min. The supernatant thus obtained was used for the measurement of endogenous GSH (Moron et al., 1979). 5,5′-Dithiobis(2-nitrobenzoic acid) (DTNB) react with the thiol (–SH) group of glutathione at pH 8.0 to produce a yellow colored ion whose concentration was measured at 412 nm. The remaining third part of the homogenate was used to estimate total protein and SOD and CAT activities. It was centrifuged at 5000 rpm for 10 min and supernatant was collected. The enzyme activity of SOD was investigated by assessing the extent of inhibition of pyrogallol autoxidation at pH 8.4 (Marklund and Marklund, 1974). The amount of protein was estimated by Bradford method, wherein the tissue protein bound to Coomassie dye of Bradford reagent under acidic conditions resulted in blue color whose absorbance was measured at 595 nm (Bradford, 1976). Lastly, CAT enzyme activity was assessed by measuring difference in H_2_O_2_ extinction per unit time, as described previously (Aebi, 1984).

### Serum CK–MB and LDH Enzymes Activities

The serum levels of CK–MB and LDH enzymes are considered as gold standard for the assessment of myocardial infarction. To determine the extent of myocardial injury, the release of intracellular LDH and CK–MB were assessed in the serum using commercially available kit as per manufacturers’ instructions.

### Estimation of Serum TNF-α Levels

To assess the levels of TNF-α, the wells of the ELISA plate were coated with a TNF-α capturing antibody. The samples and biotinylated anti-rat TNF-α antibodies were then incubated with capture antibodies for 3 h. After thorough washing, horseradish peroxidase (HRP)–streptavidin complex was added and again incubated for 20 min. A ready-to-use chromogen which acts with the bound enzyme was added to produce a colored reaction product whose absorbance was read spectrophotometrically at 450 nm (Bio Tek Instruments, USA) using reference filter at 630 nm. Simultaneously, a series of standard dilutions of TNF-α were run to obtain a standard graph. The standard curve thus obtained was used to determine TNF-α levels in the sample as the intensity of colored product is directly proportional to the concentration of TNF-α in the sample and expressed as pg/ml.

### Estimation of Serum NO Levels

Griess reagent method was used for the estimation of NO levels in the samples. The reagent was obtained from Promega Corporation, USA. Briefly, the samples and sulfanilamide solutions were added into 96-well assay plate and incubated for 20 min. Later, *N*-1-naphtylethylenediamine dihydrochloride solution was added and absorbance was read at 520 nm wavelength (Bio Tek Instruments, USA). The serum NO concentrations were determined using the standard nitrite reference curve and expressed as μmol/l (Nepal et al., 2012).

### Histopathological Evaluation

The excised heart tissues were immediately fixed in 10% neutral buffered formalin. The thick sections (5 μm) were cut using microtome and stained using hematoxylin and eosin and observed under the light microscope. At least, three hearts from each group were examined for histological examination and graded for the severity of changes using score on a scale of (-) no change; (+) focal changes; (++) patchy changes; (+++) confluent changes; (++++) massive changes. The degree of necrosis was graded and scored as described previously by a pathologist masked to the experimental groups (Malik et al., 2011b).

### Ultrastructural Evaluation

The heart tissues fixed in the Karnovsky’s solution, were washed in phosphate buffer (0.1 M, pH 7.4) and post fixed for 2 h in 1% osmium tetroxide in the phosphate buffer at 4°C. Later the tissue was embedded in araldite CY212 to make tissue blocks. Sections (70–80 nm) were cut by ultramicrotome (Ultracut E, Reichert, Austria), stained with uranyl acetateand lead acetate to visualize under transmission electron microscope (Morgagni 268D, FeiCo., The Netherlands). The investigators performing the ultrastructural and histopathological evaluation were blinded for the treatment protocol.

### Western Blot Analysis

The tissue homogenate (equal to 40 μg protein tissue) was loaded in the sodium dodecyl sulphate-polyacrylamide gel electrophoresis (SDS-PAGE) and then transferred to nitrocellulose membrane. The membrane was blocked with 5% Bovine serum albumin (BSA) for 1 h and probed with primary antibodies such as Akt, phosphorylated (p)-Akt (Ser 473), eNOS, phosphorylated (p)-eNOS (Ser 1177), NF-κB p65 (Ser 536), IKKβ (L 570), Bcl-2, Bax, and β actin (1:3000) at 4°C. The primary antibodies were detected with HRP-conjugated secondary antibodies (1:5000) after incubation for 2 h at room temperature and then protein bands were visualized by using enhanced chemiluminescence (ECL; Thermo Fischer Scientific Inc., USA) kit and quantified by densitometric analysis.

### Statistical Analysis

The data is expressed as mean ± SEM. The data from different groups were evaluated by one way analysis of variance (ANOVA) followed by *post hoc* Tukey–Kramer multiple comparison tests using the software GraphPad InStat. The criterion of statistical significance was considered statistically significant less than 0.05.

## Results

### Mortality

An overall mortality of 7.35% was observed during the study period due to bleeding following inaccurate ligation of coronary artery.

### GC–MS Analysis of SBT Pulp Oil

**FIGURE 1:**
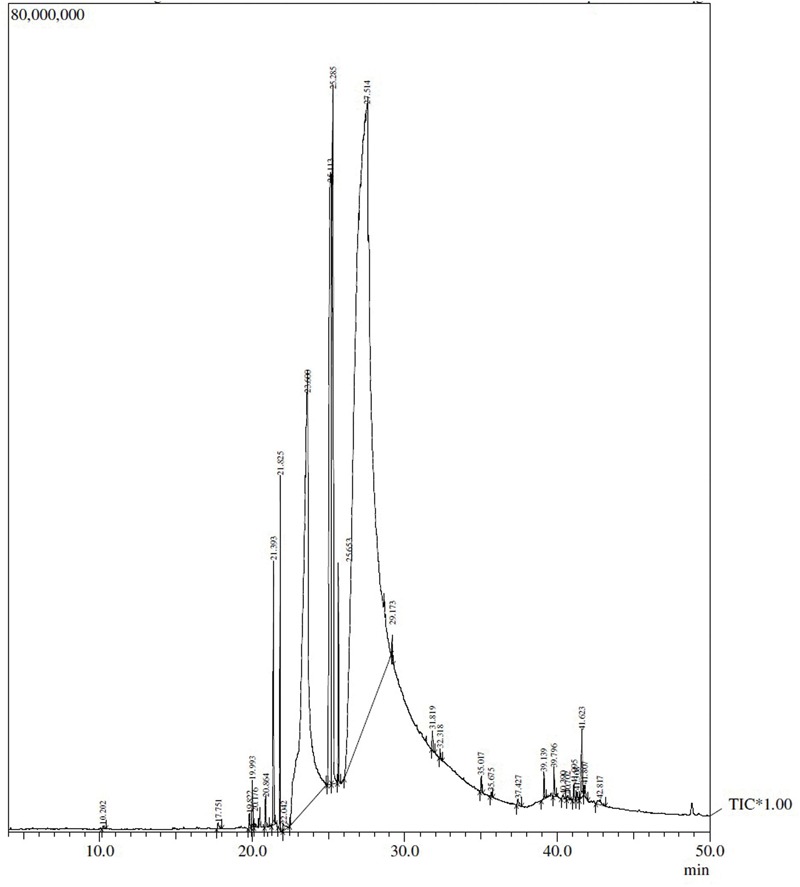
**The chromatogram of Seabuckthorn (SBT) pulp oil**.

The GC–MS analysis of SBT pulp oil performed showed structure and proportion of major components are depicted in **Figure 1** and **Table 1**.

**Table 1:** GC–MS analysis of SBT pulp oil.

R. Time	Area (%)	Compound name	Formula
21.393	1.09	9-Hexadecenoic acid, methyl ester, (Z)-; Palmitoleic acid, methyl ester	C_17_H_32_O_2_
21.825	1.06	Hexadecanoic acid, methyl ester; Palmitic acid, methyl ester	C_17_H_34_O_2_
23.600	16.69	*n*-Hexadecanoic acid; Palmitic acid	C_16_H_32_O_2_
25.113	8.35	9,12-Octadecadienoic acid (Z,Z)-, methyl ester; Linoleic acid, methyl ester	C_19_H_34_O_2_
25.285	6.44	9-Octadecenoic acid (Z)-, methyl ester; Oleic acid, methyl ester	C_19_H_36_O_2_
25.653	0.86	Octadecanoic acid, methyl ester; Stearic acid, methyl ester	C_19_H_38_O_2_
27.514	63.51	9,12-Octadecadienoic acid (Z,Z)-; Linoleic acid	C_18_H_32_O_2_

### SBT Pulp Oil Preserves Cardiac Function after IR Injury

**FIGURE 2:**
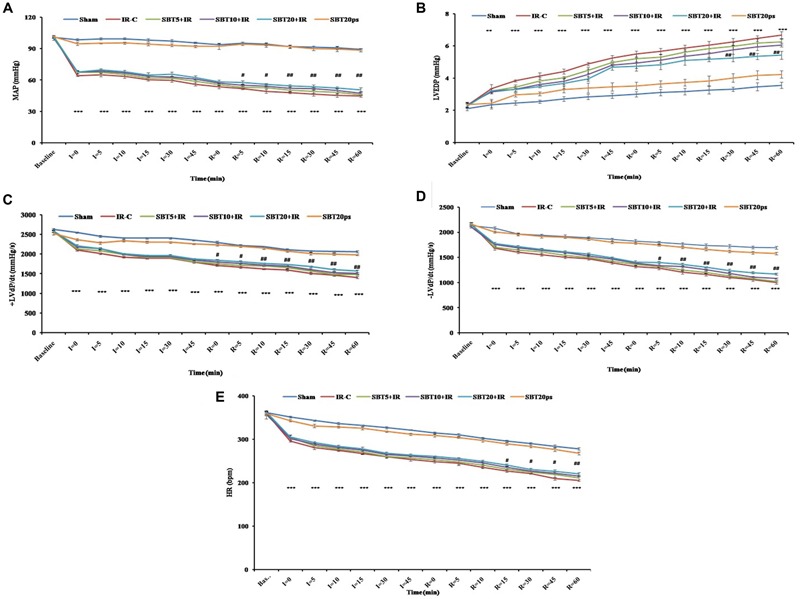
**Effect of SBT pulp oil on hemodynamic and left ventricular functions after ischemia–reperfusion (IR) injury in rats. (A)** Mean arterial pressures (MAP); **(B)** Left ventricular end diastolic pressure (LVEDP); **(C)** Maximal positive rate of the left ventricular pressure [(+LVdP/dtmax)]; **(D)** Maximal negative rate of the left ventricular pressure [(-LVd*P*/d*t*_max_)]; and **(E)** Heart rate (HR). The values are expressed as mean ± SEM; *n* = 6 in each group. ^∗∗∗^*p* < 0.001 versus sham; ^#^*p* < 0.05; ^##^*p* < 0.01 versus IR-control.

To determine whether SBT pulp oil was capable of preserving cardiac function, hemodynamic parameters such as MAP, HR, and left ventricular functions (±LVd*P*/d*t*, LVEDP) were measured in the myocardium. In IR-control group, significant decrease in MAP, HR, and ±LVd*P*/d*t* with concomitant increase in LVEDP was observed as compared to sham group (*p* < 0.001) while pretreatment with high dose SBT pulp oil significantly improved MAP (*p* < 0.01) and HR (*p* < 0.01), restored ± LVd*P*/d*t* (*p* < 0.01), and prevented rise in LVEDP (*p* < 0.01). The improvement in hemodynamic parameters was not significant with other doses of SBT pulp oil (5 and 10 ml/kg; **Figure 2**).

### Evaluation of Area at Risk and Infarct Size after IR Injury

**FIGURE 3:**
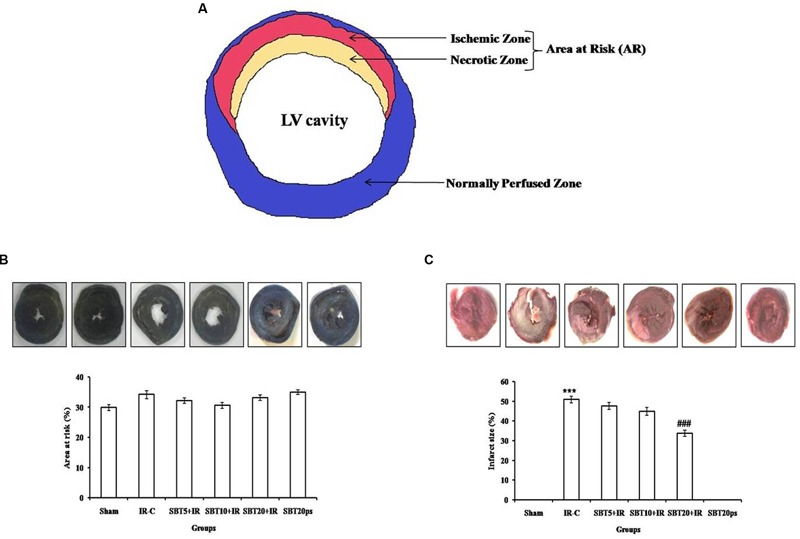
**Effect of SBT pulp oil on myocardial IR injury in different experimental groups. (A)** Representative figure; **(B)** Area at risk (%); and **(C)** Infarct size (%). IR-C: Ischemia–reperfusion control; SBT5 + IR: Seabuckthorn pulp oil 5 ml/kg/day + IR; SBT10 + IR: Seabuckthorn pulp oil 10 ml/kg/day + IR; SBT20 + IR: Seabuckthorn pulp oil 20 ml/kg/day + IR; SBT20ps: Seabuckthorn pulp oil 20 ml/kg/day *per se*. The values are expressed as mean ± SEM; *n* = 6 in each group. ^∗∗∗^*p* < 0.001 versus sham; ^###^*p* < 0.001 versus IR-control.

In our study, SBT pulp oil significantly salvaged the ischemic zone and reduced the progression of infarction over the course of time whereas in IR-control group, the infarct zone increased. This difference is seen with triphenyltetrazolium chloride (TTC) staining. However, no significant difference in mean area at risk was observed between all the groups as the site of coronary artery occlusion is maintained constant in all groups (**Figure 3**).

### SBT Oil Inhibits Lipid Peroxidation and Release of Myocardial Enzymes after IR Injury

In IR-control group, there was significant increase in level of MDA (*p* < 0.001), a lipid peroxidation byproduct was observed as compared to sham group. Furthermore, IR injury caused damage to cell membrane and releases cardiac marker enzymes from the myocardium as demonstrated by significantly elevated level of CK–MB and LDH in the serum (*p* < 0.001). SBT pulp oil dose-dependently decreased the formation of MDA (*p* < 0.05) and prevented release of CK–MB (*p* < 0.01) and LDH (*p* < 0.01) from the myocardium to serum and thus, maintained structural integrity in the myocardium (**Table 2**).

**Table 2:** The effect of SBT pulp oil on lipid peroxidation, antioxidants, cardiac injury enzymes, NO, and TNF-α level.

Groups	MDA (nmole/g tissue)	GSH (μmole/g tissue)	SOD (U/mg protein)	CAT (U/mg protein)	LDH (U/L)	CK-MB (U/L)	TNF-α (pg/ml)	NO (μmol/l)
Sham	54.81 ± 4.71	1.12 ± 0.08	3.99 ± 0.12	0.058 ± 0.006	458.20 ± 6.20	400.97 ± 6.35	20.97 ± 2.78	30.85 ± 2.14
IR-C	100.33 ± 5.29^∗∗∗^	0.64 ± 0.05^∗∗∗^	3.29 ± 0.12^∗∗∗^	0.025 ± 0.004^∗∗^	723.85 ± 9.54^∗∗∗^	676.81 ± 8.94^∗∗∗^	65.87 ± 3.46^∗∗∗^	14.88 ± 2.01^∗∗∗^
SBT5 + IR	91.47 ± 5.19	0.80 ± 0.04	3.52 ± 0.08	0.033 ± 0.004	697.16 ± 11.94	660.44 ± 12.52	59.49 ± 2.22	19.15 ± 1.79
SBT10 + IR	81.68 ± 4.98	0.89 ± 0.04	3.60 ± 0.04	0.042 ± 0.001	684.52 ± 11.68	643.48 ± 11.86	55.39 ± 3.24	21.39 ± 1.95
SBT20 + IR	78.05 ± 4.32^#^	0.98 ± 0.05^##^	3.72 ± 0.06^#^	0.051 ± 0.005^#^	669.28 ± 11.91^##^	619.54 ± 7.57^##^	49.20 ± 2.85^##^	25.01 ± 3.21^#^
SBT20ps	63.21 ± 3.32	1.07 ± 0.07	3.92 ± 0.08	0.054 ± 0.008	414.63 ± 7.45	489.92 ± 13.88	31.04 ± 2.76	28.19 ± 2.62

*IR-C, Ischemia-reperfusion control; SBT5 + IR, Seabuckthorn pulp oil 5 ml/kg/day + IR; SBT10 + IR, Seabuckthorn pulp oil 10 ml/kg/day + IR; SBT20 + IR: Seabuckthorn pulp oil 20 ml/kg/day + IR; SBT20ps, Seabuckthorn pulp oil 20 ml/kg/day *per se*. TBARS, Thiobarbituric acid reactive substance; LDH, Lactate dehydrogenase; CK–MB, Creatine kinase–MB isoenzyme; GSH, Reduced glutathione; SOD, Superoxide dismutase; CAT, Catalase. The values are expressed as mean ± SEM; *n* = 6 in each group. ^∗∗∗^*p* < 0.001; ^∗∗^*p* < 0.01 versus sham; ^#^*p* < 0.05; ^##^*p* < 0.01 versus IR-control.*

### SBT Pulp Oil Restores Antioxidants in the Myocardium after IR Injury

Ischemia–reperfusion injury resulted in oxidative stress which caused significant reduction in the activities of antioxidant enzymes SOD and CAT, and GSH content as compared to sham group (*p* < 0.01 for CAT and *p* < 0.001 for SOD and GSH). SBT pulp oil dose dependently augmented the activities of these antioxidants and attenuated the deleterious effect of IR injury on myocardium. However, the most pronounced effect was observed with SBT pulp oil (20 ml/kg; **Table 2**).

### SBT Pulp Oil Normalizes Serum NO and TNF-α Levels after IR Injury

TNF-α is one of the important cytokines in mediating inflammation while NO is known to suppress such cytokines. So, serum NO and TNF-α levels were measured to assess their role in IR injury. IR significantly (*p* < 0.001) increased serum TNF-α and decreased NO levels in comparison to sham group, which indicates marked inflammation in rats. SBT pulp oil dose dependently (20 ml/kg) decreased inflammation and caused significant reduction in TNF-α (*p* < 0.01) and increase in NO (*p* < 0.05) levels as compared to IR-control group (**Table 2**).

### SBT Pulp Oil Preserves Structural Integrity of Myocardium after IR Injury

**FIGURE 4:**
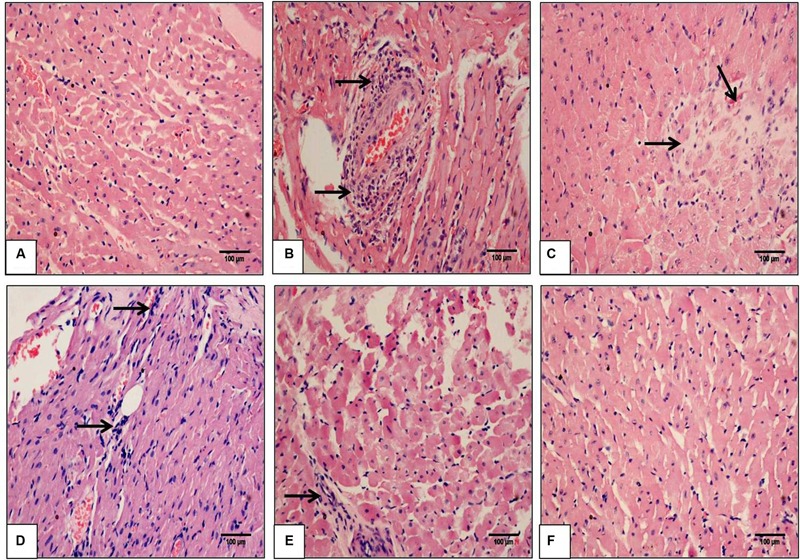
**The effect of SBT pulp oil on myocardium histoarchitecture (scale bar: 100 μm). (A)** Sham; **(B)** Ischemia-reperfusion control; **(C)** Seabuckthorn pulp oil 5 ml/kg/day + ischemia-reperfusion; **(D)** Seabuckthorn pulp oil 10 ml/kg/day + ischemia-reperfusion; **(E)** Seabuckthorn pulp oil 20 ml/kg/day + ischemia-reperfusion; and **(F)** Seabuckthorn pulp oil 20 ml/kg/day *per se*; (*n* = 3; 20X. Arrow (→) indicates necrosis and inflammation.

To visualize the extent of damage to cardiac tissue following IR injury and/or SBT pulp oil administration, tissue sections were stained with hematoxylin and eosin. In sham group, normal architecture of myocardium was observed while IR-control group exhibited marked inflammatory cell infiltrate, membrane damage, necrosis and edema in the myocardium and also, the histological injury score was markedly higher in this group as compared to sham group. In low dose SBT pulp oil (5 ml/kg) treated group, degree of histological changes were similar to the IR-control group but medium dose SBT pulp oil (10 ml/kg) group showed less inflammation and edema as compared to IR-control group. However, tissue sections of high-dose SBT pulp oil (20 ml/kg) pretreatment group showed marked reduction in myonecrosis, inflammation, and edema and exhibited a low histological injury score (**Figures 4A–F**; **Table 3**).

**Table 3:** The histopathological grading of myocardium in different experimental groups.

Groups	Necrosis	Edema	Inflammation
Sham	-	-	-
IR-C	+++	+++	++++
SBT5 + IR	+++	+++	+++
SBT10 + IR	++	++	+++
SBT20 + IR	-	-	+
SBT20ps	-	-	-

*IR-C, Ischemia-reperfusion control; SBT5 + IR, Seabuckthorn pulp oil 5 ml/kg/day + IR; SBT10 + IR, Seabuckthorn pulp oil 10 ml/kg/day + ; SBT20 + IR, Seabuckthorn pulp oil 20 ml/kg/day + IR; SBT20ps, Seabuckthorn pulp oil 20 ml/kg/day *per se*. The score represents: (-), absence of any myonecrosis, edema, and inflammation; score (+), focal areas of myonecrosis, edema, and inflammation; score (++), patchy areas of myonecrosis, edema, and inflammation; score (+++), confluent areas of myonecrosis, edema, and inflammation; score (++++), massive areas of myonecrosis, edema, and inflammation.*

**FIGURE 5:**
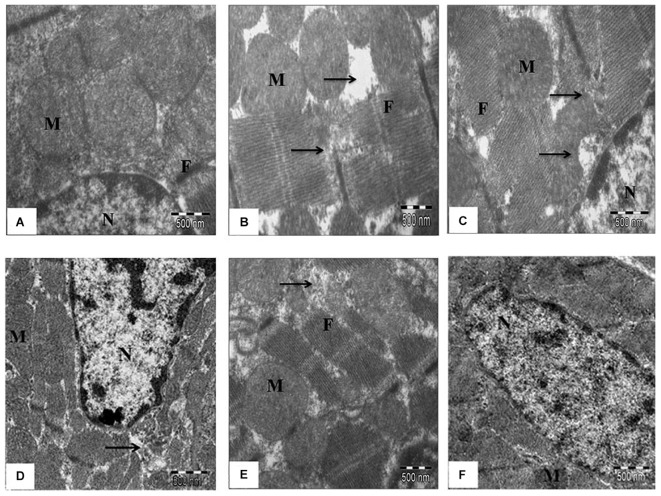
**The effect of SBT pulp oil on myocardium ultrastructure (scale bar: 500 nm). (A)** Sham; **(B)** Ischemia-reperfusion control; **(C)** Seabuckthorn pulp oil 5 ml/kg/day + ischemia-reperfusion; **(D)** Seabuckthorn pulp oil 10 ml/kg/day + ischemia-reperfusion; **(E)** Seabuckthorn pulp oil 20 ml/kg/day + ischemia-reperfusion; **(F)** Seabuckthorn pulp oil 20 ml/kg/day *per se*; (*n* = 3; M: mitochondria; N: nucleus; F: myofibrils; Arrow (→) indicates myofibrillar damage.

These findings were further confirmed by ultrastructural evaluation using TEM that showed significant myofibrillar degeneration and derangement with swollen and irregular mitochondria and distorted *Z* lines in IR-control group as compared to sham group. Similar ultrastructural changes were seen in low- and medium-dose SBT pulp oil (5 and 10 ml/kg) treatment group, although, changes were mild in medium dose pretreatment group as compared to IR-control group. On the contrary, in high dose SBT pulp oil pretreatment group, there was preservation of nuclear and mitochondrial architecture with striking absence of myofibrillar degeneration (**Figures 5A–F**). On the basis of above results; 20 ml/kg was considered as the most protective dose and further used for Western blot analysis.

### SBT Oil Mediates Cardioprotective Effects Through Activation of the Akt/eNOS Pathway

**FIGURE 6:**
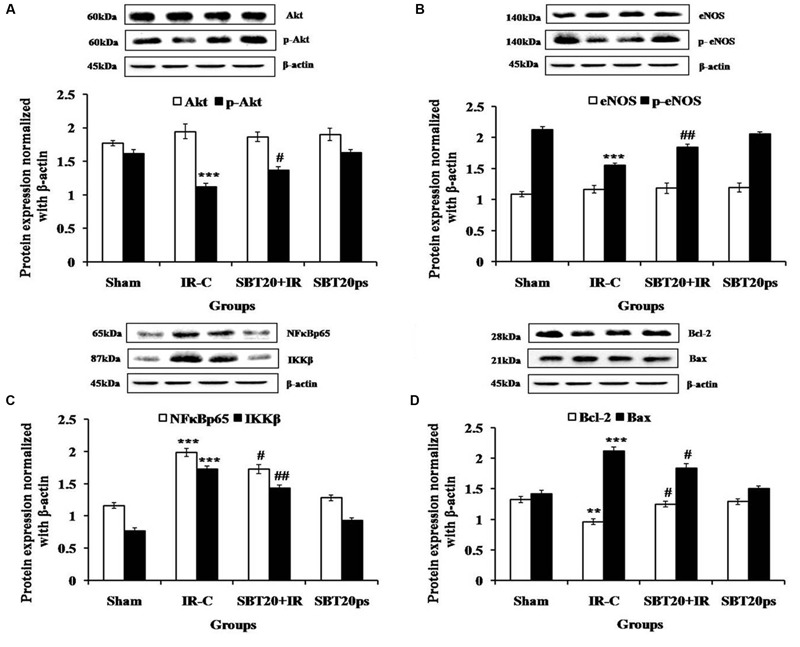
**The effect of SBT pulp oil on protein expressions in heart homogenate in different experimental groups. (A)** Akt, p-Akt; **(B)** eNOS, p-eNOS; **(C)** NF-κB p65, IKKβ; **(D)** Bcl-2, Bax. Protein expressions are normalized with beta-actin. All the values are expressed as mean ± SEM; *n* = 3 per group. ^∗∗∗^*p* < 0.001; ^∗∗^*p* < 0.01 versus sham; ^#^*p* < 0.05; ^##^*p* < 0.01 versus IR-control.

Akt induced eNOS phosphorylation at serine 1177 is an important mechanism to protect heart against IR injury (Han et al., 2012; Zhang et al., 2014). IR injury resulted in significantly decreased expressions of p-Akt (*p* < 0.001), and p-eNOS (*p* < 0.001) in the myocardium as compared to sham group. Interestingly, SBT pulp oil (20 ml/kg) treatment significantly triggered more pronounced expressions in the IR challenged myocardium in comparison to IR-control group (**Figure 6**).

### SBT Pulp Oil Down-Regulates Apoptosis and Inhibits Inflammation

To further verify the anti-inflammatory activity of SBT pulp oil, Western blotting was done on downstream proteins of TNF-α such as NF-κB and IKKβ. Further, SBT pulp oil was investigated for any effect on myocardial apoptosis. IR injury resulted in increased NF-κB (*p* < 0.001) and IKKβ (*p* < 0.001) levels with increased expression of Bax (*p* < 0.001), a marker of apoptosis and decreased expression of Bcl-2 (*p* < 0.01), an anti-apoptotic protein, in the myocardium. Pretreatment with SBT pulp oil (20 ml/kg/day) to IR-treated rats decreased downstream proteins of TNF-α such as NF-κB (*p* < 0.05) and IKKβ (*p* < 0.01) and prevented NF-κB mediated expression of inflammatory genes. It up-regulated cellular Bcl-2 (*p* < 0.05) and down regulated Bax (*p* < 0.05), which indicates that SBT pulp oil has anti-apoptotic property in its cardioprotection (**Figure 6**).

## Discussion

The present study demonstrate that systemic administration of SBT pulp oil attenuates IR injury by stabilizing hemodynamic parameters, increasing antioxidant defense system, decreasing inflammatory markers, and upregulating the anti-apoptotic proteins that were mediated by augmentation of Akt/eNOS-signaling pathway and increased NO availability and activity.

Ischemia–reperfusion injury initiates cellular events that lead to necrotic changes in the myocardium. In the present study, IR injury caused a marked decline in both contractility; [(+LVd*P*/d*t*)] and relaxation [(-LVd*P*/d*t*)], arterial pressure and heart rate along with an increase in preload; (LVEDP). Whereas, treatment with SBT pulp oil observed to dose-dependently improve cardiac performance and increase the cardiac output. Though, the exact molecular mechanism of cardioprotection elicited by SBT pulp oil is not fully known but phosphorylation of Akt/eNOS and inhibition of TNF-α/IKKβ/NF-κB is believed to be a putative mechanism for the cardioprotective effects. Previous studies have shown that Akt/eNOS phosphorylation decrease intracellular calcium overload by activating sarcoplasmic reticulum Ca^2+^ -ATPase2a (SERCA2a) and improving cardiac contractility (Braz et al., 2009; Catalucci et al., 2009; Omar et al., 2010). Furthermore, the inhibition of inflammation and cell death of cardiomyocytes *via* TNF-α/IKKβ/NF-κB pathway as well as down-regulation of pro-apoptotic proteins improves the cardiac function (Bharti et al., 2012).

On biochemical aspects, IR injury is known to increase inflammatory cytokines such as TNF-α and IL-6 that activate IKKβ/NF-κB in the myocardium (Murphy and Steenbergen, 2008). The activation of inflammatory mediators results in increased generation of reactive oxygen species (ROS) such as superoxide dismutase (O _2_^Δ-^), hydroxyl ion (OH^-^) and hydrogen peroxide (H_2_O_2_), which causes lipid membrane damage (Thiemermann, 2004). In line to this report, we also observed that increased serum TNF-α level in IR-control group exhibit correlation with increased expression of IKKβ/NF-κB in the myocardial tissues. Additionally, an increased MDA and decreased antioxidant status was also observed in myocardial tissues. Taken together, SBT pulp oil appears to strengthen the antioxidant defense system and exert anti-inflammatory activity by inhibiting TNF-α/IKKβ/NF-κB pathway.

In parallel to improved antioxidant defense, there was decreased phosphorylation of Akt/eNOS and increased expression of pro-apoptotic protein Bax in IR group. SBT pulp oil significantly increased phosphorylation of Akt/eNOS in IR injured myocardium. The phosphorylation of Akt/eNOS inhibits conformational change in Bax and prevents its translocation from cytosol into mitochondria (Kroemer et al., 1998; Kennedy et al., 1999). Further, this also increased the production of NO which inhibits platelet aggregation, ROS production, and neutrophil infiltration by decreasing the expression of adhesion molecule in the vascular endothelium. It also inhibits the opening of mitochondrial permeability transition pore (mPTP) and prevents apoptosis (Balakirev et al., 1997; Dimmeler et al., 1999). Thus, Akt/eNOS phosphorylation and inhibition of pro-apoptotic protein can be other plausible mechanisms of cardioprotection.

Apart from changes in hemodynamic and biochemical parameters, cardiac enzymes CK-MB and LDH have been used as diagnostic markers for confirmation of myocardial IR injury. The IR injury disrupts sarcolemmal integrity releasing these enzymes from damaged myocytes into the extracellular fluid (Mohanty et al., 2008; Murphy and Steenbergen, 2008). This caused increased leakage of these enzymes from cardiomyocytes to serum and this was further found to be normalized by prior treatment with SBT pulp oil. This indicates that SBT pulp oil maintains the sarcolemmal integrity, thereby restricting the leakage of the myocardial enzymes. In addition, the morphological analysis also revealed less edema, inflammation and necrosis that can be translated into myocardial salvage and rescue of the myocardial tissues following IR injury in the SBT pulp oil treatment group.

The GC–MS analysis of SBT pulp oil showed that it is rich in many important fatty acids such as linoleic acid (LA), oleic acid, palmitic acid, palmitoleic acid, and stearic acid. A study by Djoussé et al. (2001), concluded that intake of LA one of the essential omega-6 polyunsaturated fatty acid (PUFA) is associated with decreased risk of cardiovascular disease. *In vitro* studies with vascular endothelial cells have shown that LA (which is present in highest concentration in SBT pulp oil) has anti-inflammatory properties by suppressing the production of adhesion molecules, chemokines and interleukins (De Caterina et al., 2000). Many other similar studies have also shown that LA decreases cardiovascular risk factors such as inflammation, blood pressure, insulin resistance, and low density lipoprotein (LDL) and increase cardioprotective high density lipoprotein (HDL; Mensink and Katan, 1992; Grimsgaard et al., 1999; Summers et al., 2002). Another study by Ferrucci et al. (2006), also established anti-inflammatory effects of LA, as higher LA levels in plasma were associated with decreased serum pro-inflammatory cytokines. It is also reported that LA and oleic acid decrease LDL and prevent LDL oxidation that is a trigger for oxidative stress (Tsimikas et al., 1999). Thus, it can be reasonably speculated that the beneficial effect of SBT pulp oil in IR injury may be attributed to the components of pulp oil such as LA and oleic acid. Based on the findings, it can be concluded that SBT pulp oil has potential to ameliorate myocardial IR injury through phosphorylation of Akt/eNOS, inhibition of TNF-α/IKKβ/NF-κB and expression of Bax. The effect may be attributed to the essential omega fatty acids which are known to be beneficial in this condition. However, these beneficial effects of oil should be ascertained in further animal studies and translated in humans to encourage its nutritional or pharmacological usage in coronary heart disease.

## Author Contributions

All the authors provided important intellectual content, reviewed the content and approved the final version for the manuscript. Contributed significantly, read and approved the manuscript: KS, JB, SM, RKM, NG, DA, TN, SG, and SO. Conceived and designed the experiments: JB, SO, and DA. Performed the experiments: KS, SM, RKM, and NG. Performed Histopathology and electron microscopy: TN, KS, JB, SM, and RM. Analyzed the data: KS, JB, SO, SG, and DA. Contributed reagents/materials/analysis tools: SO and DA. Wrote the paper: KS, SG, JB, SO, and DA.

## Conflict of Interest Statement

The authors declare that the research was conducted in the absence of any commercial or financial relationships that could be construed as a potential conflict of interest.
