# Corrigendum: Seabuckthorn Pulp Oil Protects against Myocardial Ischemia–Reperfusion Injury in Rats through Activation of Akt/eNOS

**DOI:** 10.3389/fphar.2018.01557

**Published:** 2019-01-31

**Authors:** Kapil Suchal, Jagriti Bhatia, Salma Malik, Rajiv Kumar Malhotra, Nanda Gamad, Sameer Goyal, Tapas C. Nag, Dharamvir S. Arya, Shreesh Ojha

**Affiliations:** ^1^Cardiovascular Research Laboratory, Department of Pharmacology, All India Institute of Medical Sciences, New Delhi, India; ^2^Department of Pharmacology, R. C. Patel Institute of Pharmaceutical Education and Research, Shirpur, India; ^3^Department of Anatomy, All India Institute of Medical Sciences, New Delhi, India; ^4^Department of Pharmacology and Therapeutics, College of Medicine and Health Sciences, United Arab Emirates University, Abu Dhabi, UAE

**Keywords:** apoptosis, myocardial ischemia–reperfusion injury, inflammation, oxidative stress, seabuckthorn, lehberry, edible oil, natural products

In the original article, there was an error in Figure 5: The effect of SBT pulp oil on myocardium ultrastructure (scale bar: 500 nm,) as published. There was an error during image processing and some photographs were mistakenly placed in the article. The corrected Figure 5 appears below. The authors apologize for this error and state that this does not change the scientific conclusions of the article in any way. The original article has been updated.

**Figure 5:**
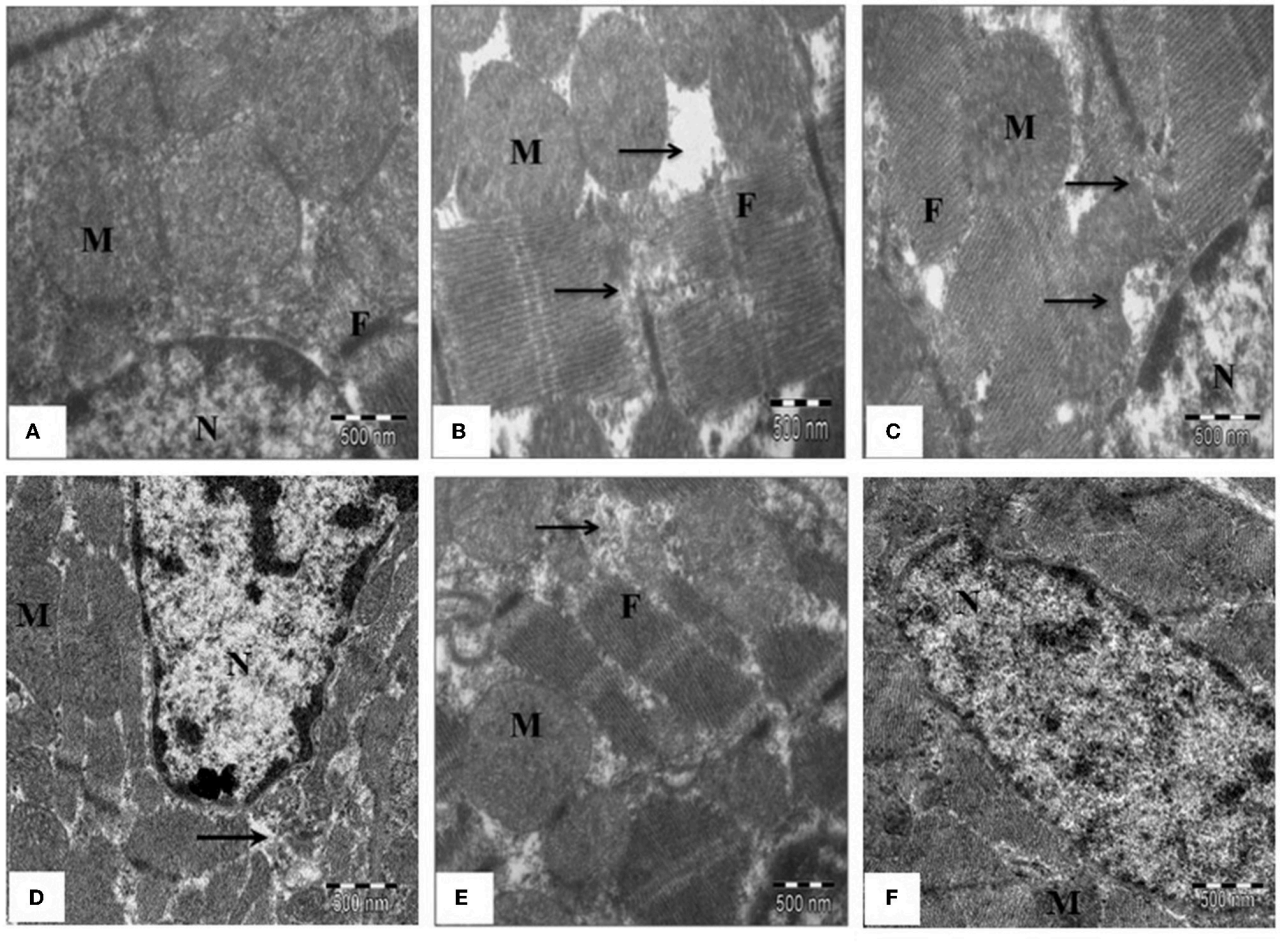
The effect of SBT pulp oil on myocardium ultrastructure (scale bar: 500 nm). **(A)** Sham; **(B)** Ischemia-reperfusion control; **(C)** Seabuckthorn pulp oil 5 ml/kg/day + ischemia-reperfusion; **(D)** Seabuckthorn pulp oil 10 ml/kg/day + ischemia-reperfusion; **(E)** Seabuckthorn pulp oil 20 ml/kg/day + ischemia-reperfusion; **(F)** Seabuckthorn pulp oil 20 ml/kg/day *per se*; (*n* = 3; M: mitochondria; N: nucleus; F: myofibrils; Arrow (→ ) indicates myofibrillar damage).

## Conflict of Interest Statement

The authors declare that the research was conducted in the absence of any commercial or financial relationships that could be construed as a potential conflict of interest.

